# Early infant diagnosis of HIV in three regions in Tanzania; successes and challenges

**DOI:** 10.1186/1471-2458-13-910

**Published:** 2013-10-02

**Authors:** Mercy G Chiduo, Bruno P Mmbando, Zahra P Theilgaard, Ib C Bygbjerg, Jan Gerstoft, Martha Lemnge, Terese L Katzenstein

**Affiliations:** 1 National Institute for Medical Research Tanga Centre, Bombo Road, Box 5004, Tanga, Tanzania; 2 Department of Infectious Diseases, University Hospital of Copenhagen, Blegdamsvej 9, Copenhagen DK-2100, Denmark; 3 Department of International Health, Immunology and Microbiology, Faculty of Health Sciences, University of Copenhagen, Øster Farimagsgade 5, Copenhagen DK-1014, Denmark

**Keywords:** Early infant diagnosis, ART, Lost to follow-up, Tanzania

## Abstract

**Background:**

By the end of 2009 an estimated 2.5 million children worldwide were living with HIV-1, mostly as a consequence of vertical transmission, and more than 90% of these children live in sub-Saharan Africa. In 2008 the World Health Organization (WHO), recommended early initiation of Highly Active Antiretroviral Therapy (HAART) to all HIV infected infants diagnosed within the first year of life, and since 2010, within the first two years of life, irrespective of CD4 count or WHO clinical stage. The study aims were to describe implementation of EID programs in three Tanzanian regions with differences in HIV prevalences and logistical set-up with regard to HIV DNA testing.

**Methods:**

Data were obtained by review of the prevention from mother to child transmission of HIV (PMTCT) registers from 2009–2011 at the Reproductive and Child Health Clinics (RCH) and from the databases from the Care and Treatment Clinics (CTC) in all the three regions; Kilimanjaro, Mbeya and Tanga. Statistical tests used were Poisson regression model and rank sum test.

**Results:**

During the period of 2009 – 2011 a total of 4,860 exposed infants were registered from the reviewed sites, of whom 4,292 (88.3%) were screened for HIV infection. Overall proportion of tested infants in the three regions increased from 77.2% in 2009 to 97.8% in 2011. A total of 452 (10.5%) were found to be HIV infected (judged by the result of the first test). The prevalence of HIV infection among infants was higher in Mbeya when compared to Kilimanjaro region RR = 1.872 (95%CI = 1.408 – 2.543) p < 0.001. However sample turnaround time was significantly shorter in both Mbeya (2.7 weeks) and Tanga (5.0 weeks) as compared to Kilimanjaro (7.0 weeks), p=<0.001. A substantial of loss to follow-up (LTFU) was evident at all stages of EID services in the period of 2009 to 2011. Among the infants who were receiving treatment, 61% were found to be LFTU during the review period.

**Conclusion:**

The study showed an increase in testing of HIV exposed infants within the three years, there is large variations of HIV prevalence among the regions. Challenges like; sample turnaround time and LTFU must be overcome before this can translate into the intended goal of early initiation of lifelong lifesaving antiretroviral therapy for the infants.

## Background

By the end of 2009 an estimated 2.5 million children worldwide were living with Human Immunodeficiency Virus -1 (HIV-1), mostly as a consequence of vertical transmission, and more than 90% of these children live in sub-Saharan Africa [1]. Without any therapeutic intervention approximately one third of HIV infected infants will die before the age of one year and half will not survive until their second birthday [2]. Studies have shown a remarkable increase in survival if HIV-infected children have access to early diagnosis and treatment [3].

HIV in Tanzania is a generalized epidemic, by early 2008 it was estimated that 1.3 million people in Tanzania mainland were living with HIV and that 10% of them were children (below 18 yrs); i.e. about 130,000 [4]. In 2007 – 2008 the HIV prevalence in the age group 15 – 49 years was reported to be 5.7% [5], which was 1.3 lower relative to the 2003–2004 survey [6]. During the past 6 years, the HIV prevalence among pregnant women has stabilized around 6% [7].

HIV can be transmitted from a mother to her child during pregnancy, at childbirth and through breastfeeding. Almost all infections in infants can be avoided by timely delivery of effective interventions to prevent mother-to-child transmission. The Tanzanian national guidelines states that all infants who are exposed to HIV should be tested for HIV-infection, even if their mothers received anti-retroviral treatment (ART) for Prevention of Mother- to- Child Transmission (PMTCT) [8]. Services included in the PMTCT program are counseling and testing of pregnant women, provision of ART for prevention of mother to child transmission of HIV, counseling and support for safe infant feeding practices, family planning and referral to Care and Treatment Centers (CTC) for the infant.

In 2004, the Tanzanian Ministry of Health and Social Welfare (MoHSW) in collaboration with stakeholders started HIV care and treatment services [8]. Within the last years there has been increasing availability of antiretroviral treatments (ART) for adults and children. However, by December 2008 only 13,400 (32%) of the 42,000 children estimated to be eligible for ART were receiving treatment [8].

In 2008 the World Health Organization (WHO), recommended early initiation of Highly Active Antiretroviral Therapy (HAART) to all HIV infected infants diagnosed within the first year of life, and since 2010, within the first two years of life, irrespective of CD4 count or WHO clinical stage [9].

The Tanzanian MoHSW in collaboration with development partners, initiated establishment of laboratory capacity and training of laboratory and Reproductive and Child Health (RCH) personnel as part of scaling up PMTCT services. Guidelines were developed for early infant diagnosis (EID) of all 4–6 weeks old HIV exposed infants using dried blood spots (DBS), and for early initiation of ART. The goal of EID of HIV is to identify HIV-infected infants early prior to the development of clinical diseases in order to provide them with life-saving treatment including ART with the aim of reducing morbidity and mortality. This is being done by collecting DBS, sending them to the zonal laboratories and results are later handled over to the parents. Four zonal laboratories located at Muhimbili National Hospital (MNH), Mbeya Referral Hospital, Kilimanjaro Christian Medical Centre (KCMC) and Bugando Hospitals were equipped to perform EID of HIV using deoxyribonucleic acid – polymerase chain reaction (DNA-PCR) testing. By June 2009 there were 507 sites providing EID services in Tanzania [8].

EID requires capacity, coordination and management of multiple health structures as well as significant logistical, financial and human investments. This raises concern regarding whether existing national programs are effective in identifying HIV exposed and HIV-infected infants and referring them to care and treatment services.

The study aim was to describe implementation of EID programs in three Tanzanian regions (Kilimanjaro, Mbeya and Tanga) with differences in HIV prevalence and logistical set-up with regard to HIV DNA testing. The completeness of testing, reporting of test results, referral and retention of infected children in the care and treatment program were investigated.

## Methods

### Study area and population

The study was conducted in Kilimanjaro, Mbeya and Tanga regions. The regions were chosen based on different HIV prevalence and access to zonal laboratory services. Kilimanjaro and Mbeya regions house their own zonal laboratories at Kilimanjaro Christian Medical Centre (KCMC) and Mbeya Referral Hospital respectively. DBS from Tanga are sent by postal couriers to KCMC for analysis. The prevalence of HIV among pregnant women is high (12.6%) in Mbeya, compared to 4.6% and 5.9% in Kilimanjaro and Tanga regions respectively [10]. Kilimanjaro region has six districts and has a total area of 13,000 sq kilometers with an estimated population of 1.5 million. Mbeya Region is administratively divided into 8 districts with an area of approx 64,000 sq kilometers and an estimated population of 2.5 million. Tanga region covers an area of 27,000 sq kilometers with an estimated population of 1.6 Million and has 8 districts.

### Ethical consideration

Access to the data was provided by the ethical clearance provided by the Tanzanian Medical Research Coordinating Committee of the National Institute for Medical Research (NIMR) and District Medical Officers of respective health facilities.

### Data collection

Data were obtained by review of the PMTCT registers from 2009–2011 at the RCH clinics and from the databases from the CTCs in all district hospitals in the three regions; Kilimanjaro, Mbeya and Tanga. For districts without a district hospital, data were collected from health facilities designated/used in place of district hospitals. All data were aggregated on monthly basis.

The following data were extracted; number of pregnant women tested for HIV during the study period (pregnant women known to be HIV positive were not retested in successive pregnancies during the study), number of women testing positive for HIV antibodies, number of deliveries (total number of infants delivered during the study period), number of exposed infants (infants born to HIV-infected mothers) registered at the RCHs, number of infants tested among the registered, number of infants with positive HIV DNA results (defined by the results of first test), as well as proportion of infected infants referred to CTCs, proportion of infants who reported to and retained at the CTCs. Age at which the infants were tested together with the date when the results were received at the RCH in order to calculate the sample turnaround time were collected. Information on referrals, treatment initiation and retention of infants to care and treatment services were obtained from the CTC databases.

### Statistical analysis

Data were entered into MS Access 2003 database and analyses were done using STATA version 11 and R version 2.15.1 statistical software packages. Exposed infants were defined as infants born to mothers confirmed to be infected with HIV, while proportion of infected infant was defined as number of HIV positive infants (single positive HIV DNA test) divided by total number of exposed infants tested for HIV infection. Sample turn-around time was calculated as the number of days from sample collection, to the date results were received at the clinic. Number of HIV positive infants was used as a denominator in calculating the proportions of the infants referred and reported to CTC as well as infants who initiated treatment and retained at the CTC. Poisson regression model was fitted to compare rates of HIV infection across the sites. Rank sum test was used to compare median age at testing and turnaround time for test results and results were reported with 95% confidence interval. A p-value < 0.05 was considered significant.

## Results and discussion

### General information

Table 1 shows the general information of the reviewed regions. Thirty-seven percent of births in Kilimanjaro region took place at the district hospitals, while in Tanga and Mbeya, 36.0% and 20.0% of pregnant women delivered at the district hospitals respectively. Tanga region was having the highest coverage of health facilities (Places providing health care including hospitals, health centers, dispensaries and clinics) with PMTCT services compared to Kilimanjaro and Mbeya; however, it had the lowest coverage of health facilities providing EID services.

**Table 1:** Distribution of births, HIV prevalence and HIV services provided in Health Facilities (HF) in the three study regions

**Regions**	**Population**	**Total number of births**	**Number of births in district hospital (%)**	HIV prevalence (95%CI) among preg. women*	**Total number of HFs**	**Number of HF with PMTCT services (%)**	**Number of HF with EID services (%)**
Kilimanjaro	1,640,087	22,646	8,478(37.4)	1.3 (95%CI: 1.10 - 1.56)	302	227 (75.2)	81/227 (36.0)
Mbeya	2,707,410	69,956	13,813(20.0)	10.4 (95%CI: 10.04–10.72)	355	269 (76.0)	169/269 (63.0)
Tanga	2,045,205	44,263	15,891(36.0)	3.8 (95%CI: 3.40 - 4.27)	270	238 (88.1)	29/238 (12.2)

*Pooled data for 2009–2011.

### Prevalence of HIV infection among pregnant women

During the period 2009 – 2011 a total of 45,399 pregnant women were tested for the first time and 3,324 (7.3%) were HIV infected. The highest prevalence of HIV infection among pregnant women was found in Mbeya 10.2%, while the lowest was observed in Kilimanjaro region (1.3%), Table 1.

### Exposed infants registered and tested at RCH clinics

During the period of 2009 – 2011 a total of 4,860 exposed infants were registered from the reviewed sites, of whom 4,292 (88.3%) were screened for HIV infection. Proportion of exposed infants tested was high (>99%) in Kilimanjaro and Mbeya, while in Tanga only 67.6% of the HIV-exposed infants were tested. Moreover, the proportion of tested infants in the three regions increased during the reviewed period from 77.2% in 2009 to 91.5% in 2010 and 97.8% in 2011. Among the infants who were tested, a total of 452 (10.5%) were found to be HIV infected (judged by the result of the first test). The prevalence of HIV infection among infants was two folds higher in Mbeya than in Kilimanjaro region (RR = 1.872, p < 0.001), Table 2. The HIV infection rate among infants was lower in Tanga region, but the difference was not statistically significant (p = 0.110). The risk of infection among the exposed infants was slightly lower in 2010 although it was not significant (p = 0.071) compared to 2009, Table 2.

**Table 2:** Risk of HIV infection between the reviewed sites

**Variable**	**Number HIV + ve (%)**	**Risk ratio**	**95%CI**	**p-value**
Region -Kilimanjaro	51 (7.47)	1		
-Mbeya	337 (13.84)	1.872	1.408 - 2.543	<0.001
-Tanga	64 (5.45)	0.737	0.508 - 1.075	0.110
Year - 2009	161 (12.64)	1		
-2010	135 (9.21)	0.808	0.641 - 1.017	0.070
-2011	156 (10.10)	0.877	0.702 - 1.097	0.250

**Figure 1:**
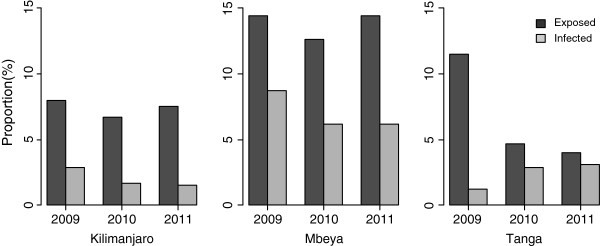
Proportion of HIV exposed and HIV DNA positive Infants in the study regions between 2009 and 2011.

Regional specific infection rates by year are as shown in Figure 1. It was observed that the proportion of exposed infants (number of exposed infants to the total number of deliveries) was decreasing in Tanga region while in the other regions it was stable. Furthermore, the proportion of HIV-DNA positive infants was decreasing in Kilimanjaro and Mbeya regions, while it was increasing in Tanga region consecutively between year 2009 and 2011.

### Age at first EID testing and turnaround time of test results

**Figure 2:**
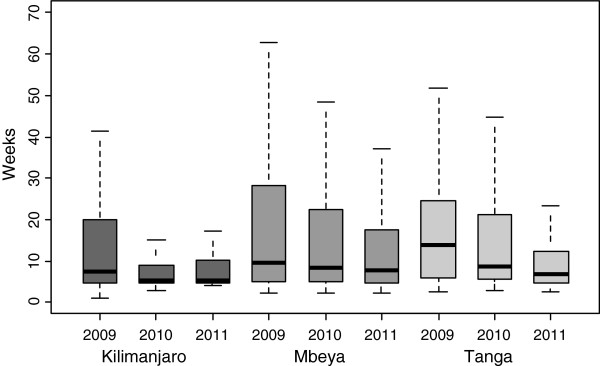
Age at first EID testing among the three regions.

The median age of infants at first EID testing was significantly higher in Mbeya (8.57 weeks) and Tanga (7.95 weeks) when compared to Kilimanjaro region (5.6 weeks), p < 0.001. Furthermore, the median ages at testing in Mbeya and Tanga regions were higher than that recommended in the EID guidelines, which is between 4 and 6 weeks. Median testing age decreased across the years in all three regions, Kilimanjaro region consistently had the lowest median testing age, Figure 2.

**Figure 3:**
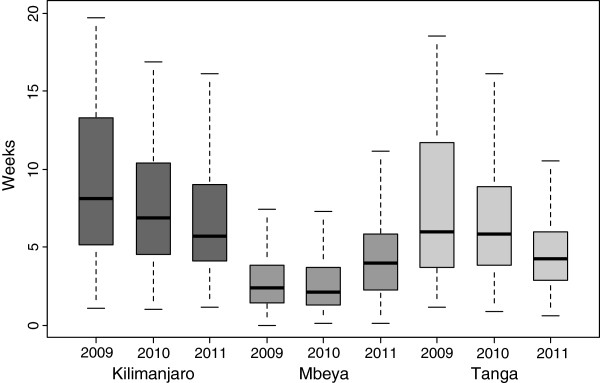
Turnaround time of results among the three regions

The proportion of mothers who returned for the test results was high within the three regions with Kilimanjaro having 99%, followed by Tanga and Mbeya with 95% and 91% respectively. Table 3, shows that the turnaround time was significantly shorter in both Mbeya (2.7 weeks) and Tanga (5.0 weeks) as compared to Kilimanjaro (7.0 weeks), p < 0,001. The turnaround time has similar decreasing pattern across the years like age at first testing for Kilimanjaro and Tanga, however in Mbeya region the turnaround time increased slightly during the study period, Figure 3.

**Table 3:** Distribution of pooled median age at testing and turnaround time for results in the study regions

	**Region**	**Median weeks**	**95%CI**	**p-value**
**Age at testing**				
	Kilimanjaro	5.9	5.6-6.4	
	Mbeya	8.6	8.0-9.0	<0.001
	Tanga	8.0	7.2-8.4	<0.001
**Turnaround time**	Kilimanjaro	7.0	6.2-7.4	
	Mbeya	2.7	2.7-2.9	<0.001
	Tanga	4.9	4.7-5.0	<0.001

### Referral and retention of infants in CTCs

Overall, the proportion of HIV infected infants referred to CTC was higher in Kilimanjaro (95.5%) and Mbeya (99.2%) than in Tanga (71.4%). Between 2009 and 2011 these proportions were almost the same (>95%) in Kilimanjaro and Mbeya, while in Tanga region, the proportion was lowest (<50%) in 2010, but increased to over 95% in 2011. Among the infants who reported to the CTC in Kilimanjaro region almost all started ART; in 2009 about 41% of the HIV infected infants reported to CTC and all of them initiated treatment, the trend was the same in 2010 and 2011, Figure 4. The figure shows that the proportion of infants reporting to CTC, initiating and being retained on treatment in Mbeya increased substantially across the years, a similar pattern was found in Tanga between 2010 and 2011.

**Figure 4:**
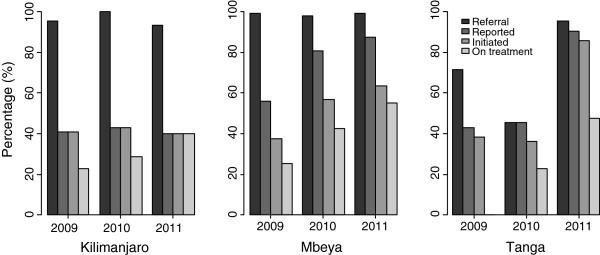
Stages of EID from RCH to CTC; proportions of HIV positive infants referred, reported to CTC, and infants who initiated treatment and retained at the CTC during 2009–2011.

Regarding retention of infants under treatment, in 2011 the proportion of infants who were still on treatment was higher than that of 2009, with Mbeya having 55.0%, Tanga with 47.6%, and Kilimanjaro 40.0% of the infants. Mbeya region consistently performed better in retaining infants under treatment. None of the infants who started treatment in 2009 were found to be on treatment during the time of review 2012 in Tanga region, while in Kilimanjaro 23.0% and in Mbeya 25.0% of the infants were still on treatment, Figure 4.

### Loss to follow –up

A substantial of loss to follow-up (LTFU) was evident at all stages of EID services in the period of 2009 to 2011. Thirty-one percent of the HIV infected infants did not report to the CTCs, Figure 4. Among the infants who were receiving treatment during the study period, 61% were basically not on treatment during the review period, Figure 4.

## Discussion

This study describes the implementation of EID program in three regions of Tanzania, with the main focus on the completeness of HIV testing, delivery of test results, referrals and retention of infected infants in the CTCs. There are four main findings from the study; first, the proportion of exposed infants being tested by DBS HIV testing increased during the study period 2009–11, second, the age at first testing and sample turn-around time decreased, third, the large variation between regions within the country and lastly the rate of loss to follow up at all stages of EID services is still high.

The study observed the number of pregnant women tested for HIV at the RCH was low compared to the number of deliveries at the district hospitals. This is due to the fact that data for HIV testing were obtained at the RCH while delivery data were obtained at the delivery wards. Furthermore, it is possible that pregnant women not tested during antenatal may have been tested during labor and delivery [8] but their results were combined to those in RCH clinics.

The prevalence of HIV among pregnant women tested was lower than that reported in the National HIV & AIDS surveillance [10]. However, in line with National Surveillance data we found that the HIV prevalence was consistently higher in Mbeya (10.2%) relative to the other two regions of Tanga with 3.8% and Kilimanjaro with 1.3%. The observed decrease in HIV prevalence maybe due to the effect of scaling-up programs on primary prevention of HIV among women of reproductive age [8]. Reports from sub-Saharan Africa has indicated a similar trend of decrease in HIV infection and these changes are attributed to scale up of prevention measures and increased awareness towards HIV infection [1].

Around 88% of exposed infants were screened with an increase in proportion of infants tested for HIV infection across the years, where the lowest rate of testing was below 70% in Tanga region. The observed high rate of testing is likely to be a result of scaling-up of PMTCT program which has been shown elsewhere to increase EID testing [11]. We found disparity in rates of testing across the sites. This could be explained by low coverage of EID services and the presence of a small number of laboratories which can perform HIV DNA-PCR analysis. For example in Tanga only 12% of HFs were providing EID services. Furthermore, unavailability of financial resources to support shipment of samples as well as remoteness of HF where DBS samples were collected could also be reasons for disparity in rates of testing.

Sample turn-around time showed great disparity between the regions, with lowest median seen in Mbeya region. With the initiation of EID there was no systemic transportation of DBS from the HF to the zonal laboratories; samples were given to the health personnel travelling to the regions where zonal laboratories were located. The circumstances were the same for the return of the results to the respected HF. In order to improve EID services financial support for transportation of DBS samples and results delivery to the health facilities was provided by President’s Emergency Plan for AIDS Relief (PEPFAR). The short turn-around time in Mbeya might be a result of the presence of the zonal laboratory where the DBS samples are analyzed; secondly the laboratory was using automated short message services (SMS) system to return the results to the health facilities. In Tanga the short turn-around time observed was most likely due to the fact that financial support for the systematic to and fro delivery of samples using postal couriers was provided. The use of SMS reporting was shown to shorten the turn-around time in a Zambian study, where SMS allowed the DBS samples results to be reported to relevant HF faster than would have been possible by courier delivery of the results [12]. Although KCMC Laboratory is in Kilimanjaro, turnaround time was longer in Kilimanjaro than in Mbeya and Tanga. This might be due to lack of systematic methods on sample transportation and results delivery. Turnaround time reported in this study was similar to those reported from another Tanzanian study which observed a turnaround time of 5–10 weeks [13] and that found in a program in Kenya [14].

Approximately 11% of the exposed infants tested HIV DNA positive on their first test. The infection rates were varying from one region to another, where Mbeya region had the highest rate relative to the other two regions, despite being well covered by PMTCT services. The results are consistent with what has been observed in other studies [13-16]. The reason for higher rates most likely a high prevalence of HIV infection among pregnant women in Mbeya (10.4%) compared to that of Kilimanjaro and Tanga, which ranged from 1 to 3%. The infection rate among HIV exposed infants may decrease with better resourced PMTCT programs and provision of more efficacious regimens [17].

Despite the proportion of parents/guardians who returned for PCR test results of their infants and referral to care and treatment services of HIV infected infant being high, the proportion of infants initiating treatment was low. There was, however, a trend of their increase in Mbeya and Tanga in the recent years. The high LTFU prior to initiation of treatment could have been caused by lack of awareness of the mothers as well as stigma [18]. Scaling up of PMTCT services with thoroughly counseling at each antenatal visit has increased awareness to the mothers which tends to improve visits to RCH of the mothers with exposed infants [10]. These results of fewer infected infants initiating treatment are similar with other studies that showed rapid increase in the number of infants tested but, only 22% to 38% of HIV infected infants initiated ART [11]. The study underlines the need of establishing systematic referral mechanisms to ensure that HIV infected infants are enrolled in ART programs. Such significant LTFU of HIV exposed infants has been noted by other programs in sub-Saharan Africa [13,15,17,19].

We found substantial LTFU at all stages of EID. The results are in accord with studies which reported that many HIV infected infants were LTFU at various stages [20,21]. Mortality, disclosure issues and fear of family and community discrimination were the most important reasons for LTFU reported from a study done in western Kenya [22]. More studies needs to be carried out to identify reasons for LTFU and ways of solving the problem.

The study has some limitations; the data used were from the district hospitals, and were of low quality in terms of missing important information like the reasons for LTFU at various stages as well as lack of data for some years which made it necessary pooling the data for analysis. The lack of linkage between RCHs, Delivery wards and CTCs makes it hard to investigate these issues thoroughly. These findings points to a significant need for improving data quality and reporting as well as means for linking data from several different sources of routinely data collection with the PMTCT program. Furthermore, the findings may be reflecting the practical management of EID in a population mostly living in areas with easy access to health facilities; hence our findings cannot be generalized.

## Conclusions

The study showed an increase in testing of HIV exposed infants within the three years. However there are still significant challenges that must be overcome before this can translate into the intended goal of early initiation of lifelong lifesaving antiretroviral therapy for the infected infants. These challenges relate to turnaround time, LTFU of infants at various stages of EID and in antiretroviral treatment programs. Identifying the reasons behind these challenges and addressing them is vital for the lives of the infants. In scaling up PMTCT programs it is of paramount importance to focus on linking the different services provided to optimize the identification, referral and retention of the infected infants at centered treatment clinics.

## Competing interests

The authors declare that they have no competing interests.

## Authors’ contribution

MC designed data capturing tools, supervised the data collection, participated in data analysis and prepared the manuscript. BM made substantial contribution to design of the study, analysis and interpretation of data, drafting and review of the manuscript. ZT, ICB and JG contributed in revising the manuscript for important intellectual content. ML contributed in supervising data collection, drafting and review of the manuscript. TK made substantial contributions in conceiving the idea of the study, interpretation of results, drafting and review of the manuscript. All authors read and approved the final manuscript.

## Pre-publication history

The pre-publication history for this paper can be accessed here:

http://www.biomedcentral.com/1471-2458/13/910/prepub
